# Effectiveness of Physiotherapy in Managing Symptomatology in Gambling Disorder Patients: A Systematic Review

**DOI:** 10.3390/healthcare11142055

**Published:** 2023-07-18

**Authors:** Pablo Carrascosa-Arteaga, Remedios López-Liria, Daniel Catalán-Matamoros, Patricia Rocamora-Pérez

**Affiliations:** 1 498—Research Team Group, University of Almeria, Carretera del Sacramento s/n, La Cañada de San Urbano, 04120 Almeria, Spain; pcarrascosaarteaga@gmail.com (P.C.-A.); rocamora@ual.es (P.R.-P.); 2 Health Research Centre, Department of Nursing, Physiotherapy and Medicine, University of Almeria, Carretera del Sacramento s/n, La Cañada de San Urbano, 04120 Almeria, Spain; 3 Culture and Technology Institute, Madrid University Carlos III, 28903 Madrid, Spain

**Keywords:** physical therapy, physiotherapy, gambling disorder, pathological gambler, behavioral addiction

## Abstract

Although the prevalence of gambling disorder (GD) and problem gambling has remained stable in recent years, the expansion of legalized gambling is considered a public health problem leading to significant personal, familial, and social impacts. This study aims to assess the effectiveness of various physiotherapy interventions on the symptoms of patients with GD. A systematic review following PRISMA guidelines was conducted in December 2022, using descriptors related to physiotherapy and GD in ten databases. Inclusion criteria were designed to identify clinical trials published in the last decade. Eight studies were identified, with a total of 357 patients, and the main variables measured were anxiety and depression symptoms, gambling craving, and gambling desire. The interventions included aerobic exercise, relaxation techniques, and non-invasive brain stimulation. Results suggest that physiotherapy may help with GD symptoms, although more research is needed to strengthen these findings. These findings highlight the potential of physiotherapy in treating GD and provide a basis for future research to better understand the effectiveness of these interventions.

## 1. Introduction

Gambling is a socially accepted form of recreation. Despite the fact that its prevalence has remained fairly stable in recent years (Great Britain, the Netherlands, Germany), the expansion of legalized gambling poses a public health problem. In 2015, the global prevalence of problematic gambling or pathological gambling ranged from 0.12% to 5.8%; on the other hand, the European prevalence ranged from 0.12 to 3.4%. However, due to a lack of help-seeking, which typically only takes place after financial, social, or family problems have already manifested, the percentage could be much higher. The variability in prevalence rates quoted is likely to be due to different methodologies for identifying gambling disorder (GD) rather than an increase in the problem [1]. Despite some variations in prevalence rates, in most European countries, there were consistent results with regard to socio-demographic characteristics: men, single or divorced, young, low level of education, belonging to an ethnic minority or born abroad, unemployed, or with a low income. According to the “European School Survey Project on Alcohol and Other Drugs” (ESPAD) report, 22% of students had gambled in the last 12 months, and 7.9% had placed bets online [2].

GD has been classified within non-substance-related addictive disorders since 2013 in the Diagnostic and Statistical Manual of Mental Disorders version 5 (DSM-5) [3] and since 2018 in the revision of the International Classification of Diseases—11 (ICD-11) [4], where it was previously included as an impulse control disorder. This change was agreed upon based on the behavioral similarities, the alteration of the reward system, and the efficacy of common treatment pathways with substance-related disorders [3,5].

DSM-5 [3] defines GD as “persistent and recurrent problematic gambling behavior leading to clinically significant impairment or distress, as indicated by the individual exhibiting four (or more) of the following in a 12-month period (where the gambling behavior is not better explained by a maniac episode): needs to gamble with increasing amounts of money in order to achieve the desired excitement; is restless or irritable when attempting to cut down or stop gambling; has made repeated unsuccessful efforts to control, cut back, or stop gambling; is often preoccupied with gambling; often gambles when feeling distressed; after losing money gambling, often returns another day to get even; lies to conceal the extent of involvement with gambling; has jeopardized or lost a significant relationship, job, educational or career opportunity because of gambling; relies on others to provide money to relieve desperate financial situations caused by gambling”. Based on the previous definition, it can be observed that the diagnostic criteria do not focus on the losses/winnings, the amounts bet, or the gambling practices. These diagnostic criteria focus on the consequences and how the game affects the person over a period of time.

The social and clinical consequences of this disorder seriously affect the person involved and their family, including bankruptcy, unemployment, domestic violence, breakup of personal relationships, suicidal ideation, and other comorbidities such as mood disorder, anxiety, or substance use disorder [6,7,8,9]. Martínez [10] cites some studies estimating that 50%, 30%, and 20% of this population also have an addiction to tobacco, alcohol, and illicit drugs, respectively. Suicidal ideation in clinical settings and in the general population is highly prevalent and is even higher when other comorbidities appear. Specifically, half of the population undergoing treatment present suicidal ideation, and around 17% have made a suicide attempt [5,11,12,13].

The socio-neuro-behavioral pattern of pathological gambling favors the concept that gambling is a socially learned behavior implemented by the individual to cope with stress, mood, and dysphoric emotions. In addition, it is well established in many studies that mood regulation (for example, anxiety) is not only one of the main reasons for gambling but also a predictor of disorder severity and relapse of GD [14].

Neurobiology affirms that craving in GD is explained by the alteration of the dopaminergic mesolimbic system, which explains the sensitivity to reward; the alteration of the orbitofrontal system, which balances benefits and losses, causing hyposensitivity to punishment; and abnormal functioning of the dorsolateral and dorsomedial prefrontal systems, which are in charge of inhibitory control and impulse regulation [15,16].

The participation of neurotransmitters in certain brain areas has influenced the development and maintenance of behavioral addictions (e.g., serotonin, opioids, or dopamine). Dopamine is the neurotransmitter that is most involved since it participates in all stages, including the initiation of addiction, maintenance, abstinence, and relapse [17,18,19].

The interventions with the most evidence compiled are psychological and pharmacological therapies, highlighting cognitive behavioral therapy, motivational interviews, and the combination of psychological and pharmacological therapy [5,20]. There are authors who advocate total abstinence from gambling, while others advocate control over gambling behavior. Currently, the latest studies involve a wide range of domains, evidencing a multidimensional recovery, so besides recovery from symptoms, aspects associated with the mental, physical, and social well-being of the patients should also be analyzed [5].

To date, there is no definitive drug treatment for GD. However, positive effects have been found with opioid receptor antagonists, which aim to reduce the desire to play and increase periods of withdrawal; selective serotonin reuptake inhibitors, which treat depressive and anxiety symptoms; and mood stabilizers, which modulate impulsive behaviors [21].

Psychosocial interventions are another therapy of choice for GD. The most successful treatments are those based on cognitive behavioral therapy, which aims for cognitive correction, decision-making/reward processing, and physical or psychological responses associated with gambling. On the other hand, there is the motivational interview that, through the verbalized intention of the desire, reasons, and need for change, presents a greater probability of behavior change [21,22]. Two meta-analyses have been found showing the efficacy of cognitive behavioral therapy [23,24] and another two for motivational interviewing [23,25].

Physiotherapy in mental health (PMH) is a specialty within physiotherapy that covers a wide spectrum of techniques aimed at improving or evaluating mental disorders, among which are addiction disorders. It is fundamentally based on the “body–mind” concept and is justified based on the symptoms that psychiatric disorders cause in the body and vice versa; likewise, treatment by the body can influence the baseline condition. The PMH posits that the body influences both establishing and maintaining mental symptoms. The impact on the body can be observed, for example, with depression or anxiety, which have a somatic musculoskeletal component. Anxiety is related to joint pain, back pain, abdominal pain, headache, or fatigue, while depression is associated with a kyphotic posture with internally rotated shoulders or decreased tone, in addition to generalized pain or fatigue [26,27]. Donaghy and Durward [28] defined the mental health physiotherapist as “the professional who offers a wide arsenal of physical treatment approaches aimed at relieving symptoms and improving quality of life. Therefore, the physiotherapist provides support in the evaluation and treatment of mental patients that is normally offered in conjunction with the prescribed pharmacological and psychotherapeutic treatment, in the context of an interdisciplinary team”.

PMH acts on the symptoms related to each disorder, such as craving, depression, or anxiety [29,30]. The tools of PMH have been studied for the purpose of addressing the symptomatology of dependence disorders for illegal substances, nicotine, or alcohol [31,32,33,34,35]. Catalán-Matamoros [36] commented on the important role that PMH can play in different addictions, such as those previously mentioned, highlighting interventions with physical exercise, basic body awareness therapy, therapeutic massage, or therapy relaxation.

GD belongs to the group of so-called “behavioral addictions”, which also include addictions to video games, the internet, mobile phones, or food, for which the efficacy of physiotherapy tools has already been studied [37,38,39].

Three relevant systematic reviews in relation to GD were found that include interventions based on physical therapy [40,41,42]. Two of them [40,41] evaluated the efficacy of non-invasive brain stimulation (NIBS). In the first review, Pettorruso [40] addressed the impact of these interventions on the decision processes related to gambling, both in healthy subjects and in people with GD. In the second, Zucchella [41] evaluated the efficacy of NIBS in addressing symptoms related to gambling, psychiatric symptoms, and behavioral symptoms related to gambling in populations with GD. Both reviews found positive effects for the interventions, although these were highly heterogeneous [40,41]. As for the third systematic review, Ribeiro [42] includes non-pharmacological treatments in a population with GD and only one study that applied therapeutic exercise.

The heterogeneity of the reviews found, both in terms of the type of studies included and the interventions used; the non-existence of a specific review of physiotherapy tools in addictions justifies the need for this systematic review.

The objective is to evaluate the effectiveness of physiotherapy procedures on the craving (main variable) and the different psychiatric and behavioral symptoms common among patients with GD. Integrating PMH into multidisciplinary treatments of mental disorders can help to carry out a more complete and comprehensive treatment together with other disciplines.

## 2. Materials and Methods

Following the PRISMA statement guidelines [43], a systematic review was carried out with studies from the last ten years (until December 2022), published in Pubmed, Embase, PEDro, Scopus, Web of Science, Cochrane Library, Psycarticles, SciELO, Psicodoc, and CINHAL. In addition, manual searches were performed for those references found in other related articles considered to be of potential interest. Successive searches were carried out until March 2023, following the same strategies to eliminate the possibility of not including studies published in that period. The review was registered in the International Prospective Register of Systematic Reviews (PROSPERO) under the registration code CRD42022374011.

The descriptors used were selected from MESH, DECS, and other keywords found in different studies on the subject. The search strategy followed is detailed in Table 1.

The PICOS methodology was followed for the subsequent reading and selection of articles:

- Participants: people diagnosed with GD;
- Intervention: procedures typical of physiotherapy in addictions;
- Comparison groups: other treatments, placebo, or control group;
- Outcomes: craving, symptoms of anxiety, depression, impulsivity, stress, quality of life, severity of gambling symptoms, and related physiological measures;
- Study design: clinical trials.

The inclusion criteria were: clinical trials from the last 10 years, written in English or Spanish, including only people diagnosed with GD that were treated with some form of physiotherapy procedure for addiction. The exclusion criteria included descriptive studies, systematic reviews, and meta-analyses. The search was narrowed to articles published in the last 10 years to obtain the most up-to-date results.

**Figure 1:**
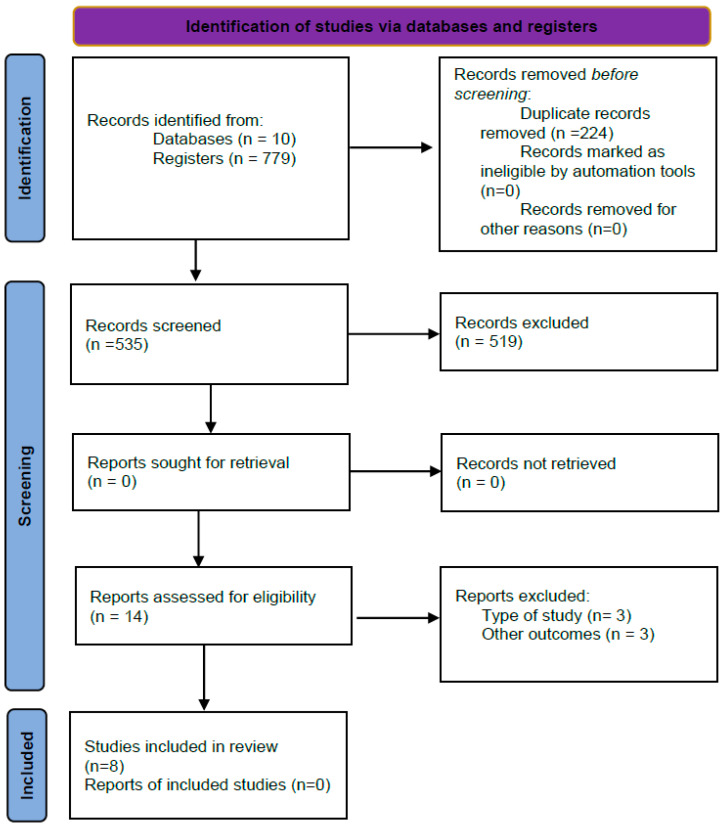
PRISMA flowchart of the article selection process.

Two independent authors (P.C.A. and R.L.L.) found 779 articles. Once duplicate studies were eliminated, and after examining the title and abstract of 535 articles, the authors read 16 full-text articles and finally selected eight studies that met the objectives and the inclusion and exclusion criteria of the review. When there were discrepancies between researchers, a third researcher (P.R.P.) was consulted. The PRISMA flowchart is shown in Figure 1.

The methodological quality of the selected studies was evaluated using the PEDro Scale [44]. In addition, the Cochrane risk of bias tool was used, following the recommendations of the Cochrane Manual for Systematic Reviews of Interventions [45,46].

## 3. Results

A qualitative analysis of the main characteristics of each study was performed, and the results are shown in Table 2.

### 3.1. Type of Study and Participants

The selected studies were randomized or non-randomized clinical trials and crossover clinical trials. A total of 357 people were included (20.22% women and 79.78% men), who were between 18 and 70 years of age. The average number of years of GD duration was between 2.5 years and 13.5 years (three studies [47,50,51] did not mention the duration of GD).

The most prevalent type of game is electronic or slot machines [9,45,47,48,49]; Sharma [51] and Zack [50] did not mention this detail. There were also some studies [9,49,50,51,52] that included subjects with different comorbidities in addition to GD.

Regarding the diagnosis in the studies, there were studies that used the DSM-4 [14,47,50,52], while others used the DSM-5 [9,48,49].

Regarding the participants, none of the studies explicitly mentioned whether they were volunteers or not. We found studies in which investigators were contacted by patients [47,48,51,52], while another mentions that participants did not seek treatment [50]. On the other hand, only one study [48] reported having provided participants with a detailed explanation of the interventions in advance. Receiving detailed information about the intervention or having the population actively seek treatment can influence the results of the study.

### 3.2. Assessment of the Main Variables

Two studies [49,52] evaluate neurobiological and physiological parameters using magnetic resonance spectroscopy (MRS) and blood tests, respectively, and attempt to relate them to the symptoms of GD. Dickler [49] evaluated the levels of GABA, Glx metabolite, and NAA in the prefrontal and striatal areas. Angelo [51] focused on the changes in cortisol, prolactin, and adrenocorticotropic hormone levels that exercise can cause, and that have an effect on the hypothalamic–pituitary–adrenal axis, leading to a positive effect on mental health. Furthermore, Sauvaget [47] evaluated heart rate (HR) and blood pressure (BP) as physiological measurements that can influence physiological arousal and craving; Zack [50] also evaluated BP.

The use of scales with a high degree of subjectivity, such as the Visual Analogue Scale (VAS) or the Visual Analogue Scale of Craving, is present in different selected trials [14,47,48,50,52]. However, the use of this type of scale is supported by self-reported scales; for example, Linardatau [14] evaluated stress using the Dass-21 Scale, Angelo [52] assessed craving with the Pennsylvania Craving Scale and the Craving Questionnaire, and Sauvaget [47] applied the Gambling Craving Scale.

All the studies carried out a baseline evaluation and a final evaluation after the intervention, but none carried out any follow-up evaluations to assess whether the effects of the interventions were maintained over time.

This review mentions the effectiveness of each intervention if statistically significant differences were found between groups in the variables studied in the results section. On the other hand, the response rate was not mentioned in the studies found, understanding that the results must last over time and, as there was a different objective for each treatment or person (reduction or gambling abstinence) it became difficult to count the response rate. In addition, adequate follow-up was not carried out for a long-term evaluation.

### 3.3. Interventions or Techniques Applied

#### 3.3.1. Aerobic Physical Exercise

Two studies [9,52] applied aerobic exercise in their interventions and examined the maximum heart rate (MHR). Both performed a group physical exercise intervention lasting 50 min. Penna [9] performed two weekly sessions at 70–85% of MHR for 8 weeks and compared the results with a control group (CG) that performed stretching. In the second case, Angelo [52] performed eight sessions at 65–70% MHR spread over 4 or 8 weeks, which was then compared to a CG without treatment.

#### 3.3.2. Non-Invasive Brain Stimulation (NIBS)

Four studies applied different types of NIBS in different brain areas: high-frequency repetitive transcranial magnetic stimulation (rTMS) [47,48], low-frequency rTMS [47], high-frequency continuous theta burst stimulation (cTBS), and transcranial direct current stimulation (tDCS). All studies perform stimulation in the dorsolateral prefrontal cortex (DLPFC), except Zack [50], who performed rTMS in the medial prefrontal cortex (mPFC), in addition to cTBS in the DLPFC. Sauvaget [47] and Gay [48] performed a single rTMS session in the DLPFC, albeit in different hemispheres, and a sham session with a 1-week interval to avoid any carryover effect; Dickler [49] applied tDCS to the right DLPFC in a single session together with another sham stimulation session with a 1-week interval; and Zack [50] performed three sessions with a 1-week interval, applying cTBS on the right DLPFC or rTMS on the mPFC group.

Sauvaget [47] only included participants with high reactivity to game cues (50% craving increase after a trigger stimulus) and after applying low-frequency rTMS (1 Hz) at the highest peak of desire (previously evaluated) for 6 min (360 impulses in total). The session was shortened to attribute the effect caused to the rTMS, and not to the natural progressive disappearance of craving. The placebo effect was performed by applying TENS to the skull.

Gay [48] performed a 20 min rTMS session on the left DLPFC at a frequency of 10 Hz, with an intensity of 110% of the resting motor threshold. The impulse trains had a duration of 3.2 s with a 10 s interval. The placebo consisted of a coil that did not apply stimulation (to improve blinding, TENS was applied to the ipsilateral supraorbital muscle in both groups).

Dickler [49] performed a 30 min session and applied tDCS by placing two 35 mm^2^ electrodes (anode placed over the right DLPFC and cathode placed over the left DLPFC). In the placebo group, the machine was only turned on at the beginning and at the end of the treatment. Four of the sixteen participants guessed which group they were in.

Zack [50], in the rTMS intervention group, set the intensity of the stimuli at 80% of the active motor threshold of the tibialis anterior and at a frequency of 10 Hz (three rTMS epoch with a 5 min interval; for each epoch, 15 pulse trains of 1 s duration and 10 s of train interval). In the cTBS intervention group, 80% of the motor threshold intensity of the first contralateral dorsal interosseous muscle was set, and the burst consisted of 3 pulses of 50 Hz repeating each train every 200 ms (three cTBS epoch to 20 s and 5 min interval). The coil of the rTMS CG was oriented perpendicular to the target area.

#### 3.3.3. Relaxation Exercises

Linardatau [14] and Sharma [51] applied Jacobson’s progressive muscle relaxation technique (PMR) (the latter also included diaphragmatic breathing in the intervention), as well as advice and education on exercise and other related aspects.

Linardatau [14] applied PMR sessions and breathing relaxation (RB) exercises to a Gamblers Anonymous group by means of a guided CD twice a day for 8 weeks. A session of 10 min of RB was applied (deep diaphragmatic breathing followed by slow prolonged exhalations) and 15 min of PMR (contractions and relaxations of different muscle groups with a sequence from bottom to top). A follow-up was carried out to verify the adequate performance of the exercises. The CG received psychotherapeutic treatment.

Sharma [51] applied PMR sessions lasting 60/90 min for 6 months in which a sequence of muscular contractions were performed for 10 s (legs, abdomen, chest, arms, and face), followed by relaxation for 20 s. There was a progression in the frequency of the sessions: the first month was four sessions/week, the second month was three sessions, the third was two sessions, the fourth was one session, the fifth was one session every 10 days, and the sixth was one session every 15 days. The CG did not receive any additional treatment.

### 3.4. Effectiveness of the Interventions in the Experimental Group Compared to the Control

#### 3.4.1. Effects of Aerobic Physical Exercise

Both Penna [9] and Angelo [52] found improvements by applying group aerobic exercise programs. Both studies evaluated the severity of GD with the Gambling Follow-up Scale Self Report Version, observing significant differences in the experimental group (EG) compared to the baseline. However, Penna [9] found that the CG had a similar benefit in reducing the severity of gambling. Also, Penna [9] observed benefits to psychiatric comorbidity, yet no benefits were obtained in relation to craving or to thoughts related to the game.

Angelo [52] found an improvement in craving, and symptoms of anxiety and depression; however, he did not observe a significant alteration in the levels of ACTH, prolactin, or cortisol.

#### 3.4.2. Effects of Non-Invasive Brain Stimulation

The effects of NIBS interventions have been highly heterogeneous. As regards craving, Zack [50] and Gay [48] observed a significant improvement in the rTMS group compared to the CG. Furthermore, Zack [50] and Dickler [49] did not observe an improvement in this variable when applying cTBS and tDCS, respectively, compared to CG. Finally, Sauvaget [47] found a significant improvement in craving in both groups, with no statistically significant differences between the rTMS group and CG.

The interventions carried out by Dickler [49] and Zack [50] did not obtain a significant reduction in impulsive behaviors.

Gay [48] had the only study that evaluated the severity of the disorder without finding statistically significant improvements.

Regarding the physiological measures of HR and BP, Sauvaget [47] did not find significant differences between groups. In contrast, while Zack [50] did find a decrease in BP in the cTBS group, but no changes were obtained in the rTMS group.

The levels of different neurotransmitters were also evaluated. Dickler [49] observed an increase in GABA levels in the right DLPFC after stimulation, which was related to reduced craving and reward seeking; otherwise, they did not find changes in the levels of N-acetylaspartic acid or glutamate. Despite not finding more changes, they did find a correlation between the levels of glutamate metabolites in the prefrontal area and GABA levels in the striatal area with risk-taking on the BART Scale. In addition, a relationship was observed between the levels of striatal NAA and the impulsiveness trait of the BIS-10 Scale. Finally, a relationship was detected between the level of craving and the striatal glutamate.

Zack [50] observed a significant decrease in psychostimulant sensations in the cTBS group. However, no differences were found in relation to subjective behavioral activation, subjective effects of treatment, or attentional control.

#### 3.4.3. Effects of Relaxation Techniques

Linardatau [14] and Sharma [51] agreed that relaxation techniques produced a reduction in anxiety and depression symptoms compared to the CG. Linardatau [14] obtained positive results in relation to stress, life satisfaction, sleep quality and routine. Sharma [51] observed beneficial effects in the IG of death anxiety, obsession, hysteria, and somatization, yet symptoms related to phobia increased after the intervention.

### 3.5. Methodological Quality of the Studies

The analysis of the methodological quality of the clinical trials included in this systematic review was carried out using the PEDro scale. The results are presented in Table 3.

In the selected studies, only one clinical trial [47] had a score greater than 6 points, qualifying as a study of good methodological quality; five clinical trials [9,14,48,49,50] obtained a score between 4 and 6; and two studies [51,52] scored below 4, qualifying as studies of low methodological quality.

### 3.6. Risk of Bias of Cochrane

**Figure 2:**
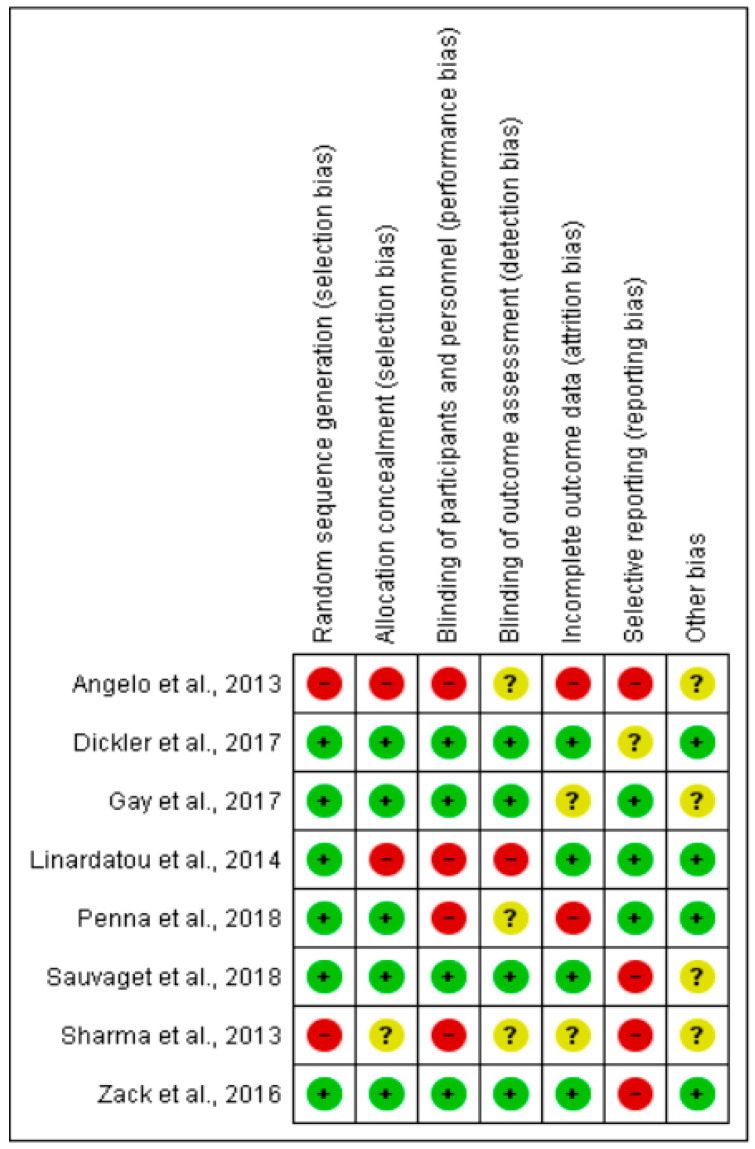
Risk of bias of the included clinical trials. +: low risk; ?: unclear risk; -: high risk [9,14,47,48,49,50,51,52].

All the included clinical trials presented some item that indicated a risk of bias, or did not show sufficient data to conclude the risk (Figure 2). Only two studies [51,52] did not perform randomization during the selection process. In addition, aspects with a greater bias in the trials included the blinding of the participants and the therapists, and the notification bias.

## 4. Discussion

This systematic review highlights the possible positive results of various PMH methods in treating addiction, including aerobic exercise, non-invasive brain stimulation (NIBS), and relaxation exercises, among patients with gambling disorder (GD). All the participants were diagnosed with GD using the DSM VI [14,47,50,52], DSM VI-TR [48], DSM V [9,44], or the diagnosis of GD was specified, even though the evaluation method used for it was not explicit [51]. The majority of the clinical trials analyzed in this review have reported positive outcomes across different symptoms associated with GD, such as reducing the desire to gamble [47,48,50,52], alleviating depressive or anxious symptoms [14,51,52], decreasing the severity of gambling problems [9,52], and increasing GABA levels in the dorsolateral prefrontal cortex (DLPFC), which is inversely related to craving and reward-seeking [49]. However, the studies did not find significant differences in variables such as compulsive behavior [48,49,50]. Moreover, none of the studies reported any significant adverse effects, except for Sharma [51], who found an increase in phobia-related symptoms after applying an intervention based on relaxation exercises. These findings have important implications for healthcare professionals in designing and implementing effective physiotherapy interventions for individuals with GD.

The studies of Penna [9] and Angelo [52] were similar in terms of duration, exercises performed, and number of sessions. However, they differed slightly in the intensity of the exercises (70/85% of MHR [9]; 65/70% of MHR [50]) and the variables evaluated. The effects of aerobic exercise have been evaluated with psychological and physiological variables in other addictions, but in the population with GD there is only one pilot study prior to the two mentioned. Angelo [53] obtained improvements in the severity of gambling symptoms (including craving, frequency, spending, and emotional distress) and in psychosocial functioning after group aerobic exercise. As for other variables considered in the literature, a study in a population with substance abuse was found, raising the importance of attending to the preferences for exercise and how it is performed by each patient to reduce dropouts and achieve greater adherence to treatment [54]. Furthermore, Penna [9] and Angelo [52] performed group interventions, so the social effect may have influenced the results. Another aspect that could have influenced the results is that the CG of Angelo [52] was comprised of people who had refused to belong to the EG, so the motivational component may have had an influence.

The four studies that applied NIBS were very heterogeneous in terms of study design, outcome measures, the cortex area in which stimulation was directed, frequency level, the number of sessions, and treatment duration. The NIBS and its effect on a population with behavior or substance addiction is a tool that still does not have a clear application protocol. These techniques try to inhibit or excite neuronal activity. Generally, low-frequency rTMS (<5 Hz) and cTBS inhibit activity in the area where they are applied, whereas high-frequency rTMS (>5 Hz) and tDCS increase excitability [40,41]. None of the clinical trials applied the same type of stimulation to the same cortex area; therefore, the effects found in this population cannot be compared. However, guidelines on the rTMS application were found that confirm an evidence level A (definitive efficacy) in the application of high-frequency rTMS in left DLPFC for depression; and evidence level B with the application of low-frequency rTMS in the right DLPFC in depression [55]. Moreover, guidelines with evidence level B were found for the application of anodal tDCS in the left DLPFC (with right orbitofrontal cathode) in major depressive episodes without drug resistance; and anodal tDCS in right DLPFC (with left DLPFC cathode) in addiction/craving [56]. These guidelines could be extrapolated to subsequent studies in the population with GD. 

Two studies [14,51] applied PMR relaxation, although there were differences in the duration of the sessions and the interventions. Linardatau [14] also performed RB. In both studies, anxiety and depression improved after the intervention, although they did not use the same evaluation scales. Linardatau [14] based his intervention on following a guided CD, so there was no real supervision of the exercises, there was no feedback from the patient after the exercises, and there was no social component of the group intervention. As for Sharma [51], the study did not specify whether it was an individual or group intervention, which could influence the results.

No previous systematic reviews were found on the different interventions that can be applied from physiotherapy to people with GD. However, one review including psychological treatments [42] in GD was found, as well as one single study applying therapeutic aerobic exercise [9]. This review [42] includes different non-pharmacological interventions, among which cognitive behavioral therapy and cognitive therapy stand out. Psychological therapies have a long history in the study of addictions, becoming one of the first-line treatments [5,20]. This study had more solid conclusions due to the large number of studies, the number of patients involved, the course of the therapies applied, and the inclusion of studies that follow-up for up to 9 years. There are different variables that are included both in this review and in that of Ribeiro [42], such as anxious or depressive symptoms or impulsivity. This can confirm that both disciplines could work together for the same objective.

Regarding the use of NIBS, two systematic reviews from 2020 [41] and 2021 [40] in the population with GD were found (they included case reports, feasibility studies, and case series). These studies applied rTMS or tDCS with a healthy population [40], and with other substance dependence populations [40,41]. Their results coincide with the present systematic review in terms of the heterogeneity in relation to the studies included, the parameters, and the area of the stimulation application, which makes it difficult to compare the results to perform a meta-analysis.

The methodological quality was evaluated with the PEDro Scale. The most consistent items were “the results of comparisons between groups” and “point measures of variability”. In contrast, the items that were least fulfilled were “blinded therapists” and “adequate follow-up”.

The Cochrane risk of bias tool shows that, due to the nature of physiotherapy interventions for addictions, it is difficult to blind the participants since, as Linardatau [14] comments, they cannot be prevented from knowing the intervention being performed by the other group. Nevertheless, three trials that applied NIBS [47,48,49] performed an intervention with a placebo, successfully blinding between 60% [46] and 77.3% [48] of the sample. While Zack [50] did not evaluate blinding, the study affirms that the process was effective in the absence of significant effects on the results. In relation to the risk of bias due to attrition, only four studies make explicit the dropouts (Penna [9], 11 dropouts; Linardatou [14], 3 dropouts; Sauvaget [47], 1 dropout; and Dickler [49], 2 dropouts), although only two mentioned the causes [14,49]. Penna [9] and Sauvaget [47] examined the results using an intention-to-treat analysis; Dickler [49] did not include the results of dropouts in the analyses; and Linardatau [14] did not mention any procedure from the analysis of dropouts. In relation to gender, a difference was observed in the population of the studies (70.85% men and 29.15% women), which can bias the applicability of the results.

While other reviews have included the GD population with other addictions or a healthy population, this is the first systematic review that addresses the applicability of physiotherapy in addictions in a population with GD. Other clinical trials regarding other behavioral addictions were not included in this review as there are already specific systematic reviews addressing other behavioral addictions in which different physical therapy tools are applied, such as exercise in internet addiction [37] or smartphone addiction [38]; or as tDCS in food-addiction [39]. Only clinical trials were included, which reduces the number of studies but increases the level of evidence. The extensive databases consulted, the manual search in the bibliography of possible studies, and the recent date of publication mean that the results found are as up-to-date as possible.

The limitations coincide with those of previous systematic reviews [40,41,42], such as the heterogeneity of the interventions and the methodological variability of the same intervention. In addition, the large number of different dependent and independent variables presented by the different studies and the design characteristics of each study gives rise to a wide variety of significant and non-significant results that are difficult to compare between studies. These factors make it difficult to draw any definitive conclusions, and a high notification bias [47,49,50,51,52] complicates performing a meta-analysis. No studies were found in which only physiotherapy tools were applied, as physiotherapy in GD is considered a complementary treatment to pharmacological and psychotherapeutic interventions in this population (and not a substitute treatment), and also because the physiotherapy intervention focuses on the symptomatology of GD and not the disorder itself.

On the other hand, we must not forget the phenomenon of natural recovery that affects this type of disorder. It has been observed in two studies based on surveys collected by Slutske [57] that mention that a large majority of people who suffer from GD throughout their lives do not become chronic, and eventually, remission or recovery arrives. This study justifies the existence of natural recovery because, according to these studies, 36% to 39% of people with a history of GD did not experience any problem related to GD in the previous year; and, only between 7% and 12% had sought treatment. This effect has also been studied in other addictions such as alcohol addiction reaching the same conclusion. This concept suggests that the results found may be influenced by this phenomenon [58]. 

Blaszczynski [59] considered that the premise of GD being an addiction may be premature. In this study, it is justified that in the absence of any comorbid psychiatric disorder, such as manic depression or schizophrenia, that causes affective disturbance and/or impairs cognitive processes, gambling represents an informed choice with responsibility for making that choice remaining with the individual. However, this review has taken into account the recent inclusion of GD as a behavioral addiction in the DSM-V and ICD-11.

Future studies should consider collecting data on the number of dropouts during the intervention, including the reasons for dropouts, and analyzing the results using an intention-to-treat methodology. Some studies have reported a high dropout rate among individuals with GD, and understanding the reasons behind this can help in developing more effective interventions that can be sustained over the long term [60]. Long-term follow-up of study participants is also necessary to determine whether the positive effects of physiotherapy interventions are maintained over time. Additionally, investigating the efficacy of physiotherapy interventions in other types of addictions, such as sexual addictions [61], could provide valuable insights into the broader potential of this approach. Finally, another point to consider is evaluating which interventions are most promising in this population and in relation to which variables to obtain a clearer and more significant conclusion to apply the intervention to a specific GD variable.

In summary, this systematic review has demonstrated the potential of physiotherapy interventions in addressing addiction-related symptoms in patients with GD. The findings suggest that physiotherapy, including aerobic exercise, NIBS, and relaxation exercises, may have a positive impact on a range of symptoms associated with GD. However, given the limitations identified in the methodological quality of the studies reviewed, caution is warranted in drawing definitive conclusions. Therefore, there is a need for further high-quality studies to evaluate the efficacy of physiotherapy interventions in this population, to better understand the mechanisms of action, and to identify the most effective approaches so that physiotherapists can apply these interventions in the health system according to scientific evidence. Such studies could provide valuable insights and evidence-based recommendations for healthcare professionals in developing more targeted and effective interventions for individuals with GD.

## 5. Conclusions

In conclusion, the field of physiotherapy for addiction provides a range of interventions that can assist in an interdisciplinary or transdisciplinary approach to address the needs of individuals with GD. This systematic review has highlighted several clinical trials that pose the positive effects of physiotherapy interventions, including therapeutic physical exercise, relaxation techniques, and non-invasive brain stimulation, on a range of psychiatric and behavioral symptoms associated with GD, including craving. Despite these promising findings, further research is needed, particularly randomized controlled trials with larger sample sizes and better methodological quality, to confirm the efficacy of these interventions and to provide evidence-based guidance for healthcare professionals. Overall, this review underscores the potential of physiotherapy in the management of GD and the importance of ongoing research in this area.

## Figures and Tables

**Table 1:** Search strategy in the different databases.

Databases			Search Terms	Results	Select Articles
PUBMED	Gambler Gambling	AND	Physiotherapy	37	4
			Physical therapy		
			Physical therapy modalities		
			Physical activity		
			Exercise		
			BBAT		
			Basic body awareness therap		
			Psychomotor therapy		
			Massage		
			Transcranial direct current stimulation Transcranial magnetic stimulation		
			Electric stimulation therapy		
			Relaxation		
			Relaxation therapy		
			Yoga		
EMBASE	Gambling Gambler	AND	Physiotherapy	22	5
			Exercise		
			Physical activity		
			Massage		
			Transcranial direct current stimulation		
			Transcranial magnetic stimulation		
			Electric stimulation therapy		
			Yoga		
			Relaxation		
PEDro	Gambling Gambler			2	1
Scopus	Gambler Gambling	AND	Physiotherapy	241	5
			Physical therapy		
			Physical therapy modalities		
			Physical activity		
			Exercise		
			BBAT		
			Basic body awareness therapy		
			Psychomotor therapy		
			Massage		
			Transcranial direct current stimulation Transcranial magnetic stimulation		
			Electric stimulation therapy		
			Relaxation		
			Relaxation therapy		
			Yoga		
Web Of Science	Gambler Gambling	AND	Physiotherapy	335	5
			Physical therapy		
			Physical therapy modalities		
			Physical activity		
			Exercise		
			BBAT		
			Basic body awareness therapy		
			Psychomotor therapy		
			Massage		
			Transcranial direct current stimulation Transcranial magnetic stimulation		
			Electric stimulation therapy		
			Relaxation		
			Relaxation therapy		
			Yoga		
Cochrane Library	Gambler Gambling	AND	Physiotherapy	59	6
			Physical therapy		
			Physical therapy modalities		
			Physical activity		
			Exercise		
			BBAT		
			Basic body awareness therapy		
			Psychomotor therapy		
			Massage		
			Transcranial direct current stimulation		
			Transcranial magnetic stimulation		
			Electric stimulation therapy		
			Relaxation		
			Relaxation therapy		
			Yoga		
Psycarticles	Gambler Gambling	AND	Physiotherapy	1	-
			Physical therapy		
			Physical therapy modalities		
			Physical activity		
			Exercise		
			BBAT		
			Basic body awareness therapy		
			Psychomotor therapy		
			Massage		
			Transcranial direct current stimulation		
			Transcranial magnetic stimulation		
			Electric stimulation therapy		
			Relaxation		
			Relaxation therapy		
			Yoga		
Scielo	Gambler Gambling	AND	Physiotherapy	2	-
			Physical therapy		
			Physical therapy modalities		
			Physical activity		
			Exercise		
			BBAT		
			Basic body awareness therapy		
			Psychomotor therapy		
			Massage		
			Transcranial direct current stimulation		
			Transcranial magnetic stimulation		
			Electric stimulation therapy		
			Relaxation		
			Relaxation therapy		
			Yoga		
Psicodoc	Gambler Gambling	AND	Physiotherapy	0	-
			Physical therapy		
			Physical therapy modalities		
			Physical activity		
			Exercise		
			BBAT		
			Basic body awareness therapy		
			Psychomotor therapy		
			Massage		
			Transcranial direct current stimulation		
			Transcranial magnetic stimulation		
			Electric stimulation therapy		
			Relaxation		
			Relaxation therapy		
			Yoga		
CINAHL	Gambler Gambling	AND	Physiotherapy	80	2
			Physical therapy		
			Physical therapy modalities		
			Physical activity		
			Exercise		
			BBAT		
			Basic body awareness therapy		
			Psychomotor therapy		
			Massage		
			Transcranial direct current stimulation		
			Transcranial magnetic stimulation		
			Electric stimulation therapy		
			Relaxation		
			Relaxation therapy		
			Yoga		

**Table 2:** Main results of the included clinical trials.

Author, Year	Type of Study Population (m/f)	Intervention	Outcomes (Outcomes Instruments)	Results
Penna, 2018 [9]	RCT n = 59 (34/25) IG: 32 CG: 27	IG: 10’ stretching and 40’ running at 70–85% MHR CG: 50’ stretching 2 times/week, 8 weeks.	- Gambling disorder severity (Gambling follow-up scale, self-report version); - Psychiatric comorbidities (Mini International Neuropsychiatric Interview); - Craving and thoughts related to gambling (Gambling Symptom Assessment Scale).	There was a significant improvement in relation to the severity of the gambling disorder (*p* = 0.01) and psychiatric comorbidities in both groups (IG: *p* = 0.005; CG: *p* = 0.015).
Sauvaget, 2018 [47]	CCT n = 31 (27/4) G1: 15 G2: 16	G1: low-frequency rTMS/sham rTMS G2: sham rTMS/low-frequency rTMS 2 sessions of 6’ (1 active rTMS session and 1 sham rTMS session) over the right DLPFC with a 1–2 weeks interval.	- Craving (VAS, Gambling Craving Scale); - Physiological measurements (HR and BP).	After rTMS sessions, a significant craving decrease was found when active rTMS (*p* < 0.01) and sham rTMS (*p* < 0.01) were applied. There were no statistically significant differences between applying active rTMS or sham rTMS (*p* = 0.18).
Gay, 2017 [48]	CCT n = 22 (14/8) G1:11 G2:11	G1: HF rTMS/sham rTMS G2: sham TMS/HF rTMS 2 sessions of 20’ (1 session of active rTMS and 1 of sham rTMS) over the left DLPFC with a 1-week interval.	- Severity of the gambling disorder (Yale–Brown Scale for Obsessive Compulsive Disorder Adapted to Pathological Gambling (GD-YBOCS)); - Craving (VAS).	There was a craving decrease after active rTMS (*p* = 0.04). Nevertheless, no significant changes were found regarding the behavior of the game (*p* = 0.68).
Dickler, 2017 [49]	CCT n = 18 (11/7) G1:18	G1: 2 sessions of 30’ (1 active session and 1 sham session) tDCS over the right DLPFC with a 1-week interval.	- Risk taking (BART); - Impulsivity trait (BIS-10); - Craving (VAS); - GABA, NAA, and Glx (MRS).	Significantly higher GABA levels were found in DLPFC after tDCS than sham stimulation in the right DLPFC (*p* = 0.039); but there were no significant differences regarding Glx (*p* = 0.733) and NAA (*p* = 0.779) in the right DLPFC. There were no significant differences in the right striatum for GABA (*p* = 0.072), Glx (*p* = 0.839) and NAA (*p* = 0.222). A positive correlation was observed between risk-taking and prefrontal Glx (*p* = 0.050) and striatal GABA (*p* = 0.045); secondly, a correlation was found between the impulsiveness and the striatal NAA (*p* = 0.036); and finally, there was a positive correlation between the craving and the striatal Glx (*p* = 0.045).
Zack, 2016 [50]	RCT n = 9 (9/0) IG1: 3 IG2: 3 GC: 3	IG1: HF rTMS over mPFC IG2: HF cTBS over right DLPFC CG: sham stimulation over mPFC 3 sessions each group with a 1-week interval.	- Craving (VAS); - Subjective behavioral activation (POMS-sf); - Psychostimulant sensations (ARCI) - Subjective effects of treatment; - Risky decision-making and game speed (trading machine); - Medication effects (Side effects checklist) - Impulsivity and attentional control after the game (Stroop task and delayed discounting task).	The craving decreased for the rTMS group (IG1) compared to the sham stimulation (CG) (*p* = 0.032); secondly, there were no improvements in the cTBS group (IG2) compared to the sham group (CG) (*p* > 0.07). The ARCI of the cTBS group (GI2) decreased significantly compared to the sham group (CG) (*p* = 0.014); there were no differences for the rTMS group (GI1) compared to the sham group (CG) (*p* > 0.10). Diastolic BP decreased significantly for the cTBS group (GI2) compared to the sham group (CG) (*p* = 0.007).
Linardatau 2014 [14]	RCT pilot n = 45 (42/3) IG: 23 GC: 22	IG: RB and PMR + education on diet, exercise, and stress (PMR and RB twice/day 25 min session) CG: diet, exercise, and stress education 8 weeks.	- Symptoms of depression, anxiety, and stress (Dass-21); - Routine (self-report Likert scale); - Life satisfaction (self-reported); - Stress (VAS); - Quality of sleep (self-reported).	A statistically significant improvement was obtained in the IG on symptoms of stress (*p* < 0.01), anxiety (*p* < 0.01), and depression (*p* < 0.01) in the Dass-21. There was also a significant improvement in the IG on self-reported stress (*p* < 0.01), in life satisfaction (*p* < 0.01), and an improvement in quality of sleep (*p* < 0.01) and daily routine (*p* < 0.01).
Sharma, 2013 [51]	CT n = 110 IG: 55 GC: 55	IG: advice and PMR (begins with 4 times/week and ends with 1 time every 15 days), 60’/90’ in duration CG: no treatment 6 months.	- Symptoms of anxiety, obsession, phobia, somatization, depression, and hysteria (Middlesex Hospital Questionnaire); - Death anxiety (Death Anxiety Scale);	The IG significantly improved differences on the Death Anxiety Scale (*p* = 0.01) and on symptoms of anxiety (*p* = 0.01), depression (*p* = 0.01), obsession (*p* = 0.01), somatization (*p* = 0.01), and hysteria (*p* = 0.01); however, phobia worsened (*p* = 0.01).
Angelo, 2013 [52]	CT n = 63 (38/25) IG: 33 GC: 30	IG: 10’ stretching and 40’ running at 65–70% HR CG: no intervention 8 sessions in 4 or 8 weeks.	- Cynical status (Gambling Follow-up Scale, Self-Reported Version); - Depression (BDI); - Anxiety (BAI); - Craving (PCS); - Craving, intensity of craving, and ability to resist urges (CQ); - Craving (Visual Analogue Scale of Craving); - ACTH, cortisol, and prolactin (blood test).	Statistically significant improvements were found in the IG regarding the psychiatric comorbidities of depression (*p* = 0.015) and anxiety (*p* = 0.026), and in the severity of the GD (*p* = 0.042). A craving reduction (on the Visual Analogue Scale of Craving) was also observed in the IG after each physical activity session (*p* = 0.003) and at the end of the program in relation to the last 24 h (*p* < 0.001) and the last 7 days (*p* < 0.001). A positive correlation was observed between before and after session anxiety with BAI (*p* = 0.004), PCS (*p* < 0.001), and CQ (*p* < 0.001).

ACTH: adrenocorticotropic hormone; ARCI: Addiction Research Center Inventory; BAI: Beck Anxiety Inventory; BART: Balloon Analogue Risk Task; BDI: Beck Depression Inventory; BISS-10: Barratt Beck Anxiety Inventory; impulsivity; BP: blood pressure; CCT: crossover clinical trial; CG: control group; CQ: Craving Questionnaire; CT: clinical trial; cTBS: continuous theta burst stimulation; DASS-21: Depression, Anxiety and Stress Scale; DLPFC: dorsolateral prefrontal cortex; GABA: γ-aminobutyric acid; Glx: glutamate; HF: high frequency; HR: heart rate; IG: intervention group; M/f: male/female; MHR: maximum heart rate; mPFC: medial prefrontal cortex; MRS: magnetic resonance spectroscopy; n: population; NAA: N-acetylaspartic; PCS: Pennsylvania Craving Scale; PMR: progressive muscle relaxation; POMS-sf: profile of mood states, short form; RB: relaxation breathing; RCT: randomized clinical trial; rTMS: repetitive transcranial magnetic stimulation; tDCS: transcranial direct current stimulation; VAS: visual analogue scale.

**Table 3:** Evaluation of the methodological quality of the articles included with the PEDro Scale.

Item PEDro Scale	1	2	3	4	5	6	7	8	9	10	11	Total Score
Penna et al., 2018 [9]	X	X	X	NO	NO	NO	X	NO	X	X	X	6/10
Sauvaget et al., 2018 [47]	X	X	X	NO	X	NO	X	NO	X	X	X	7/10
Gay et al., 2017 [48]	X	X	X	NO	X	NO	X	NO	NO	X	X	6/10
Dickler et al., 2017 [49]	X	X	X	NO	X	NO	X	NO	NO	X	X	6/10
Zack et al., 2016 [50]	X	X	X	NO	X	NO	X	NO	NO	X	X	6/10
Linardatou et al., 2014 [14]	X	X	X	X	NO	NO	NO	NO	NO	X	X	5/10
Sharma et al., 2013 [51]	NO	NO	NO	NO	NO	NO	NO	NO	NO	X	X	2/10
Angelo et al., 2013 [52]	X	NO	NO	X	NO	NO	NO	NO	NO	X	X	3/10

X = the criteria is satisfied; NO = the criteria is not specified.

## Data Availability

Not applicable.
